# *TRIP11-PDGFRB* fusion in a patient with a therapy-related myeloid neoplasm with t(5;14)(q33;q32) after treatment for acute promyelocytic leukemia

**DOI:** 10.1186/s13039-014-0103-6

**Published:** 2014-12-23

**Authors:** Hoon-Gu Kim, Ja-Hyun Jang, Eun-Ha Koh

**Affiliations:** Departments of Internal Medicine, Gyeongsang National University School of Medicine, 79 Gangnam-ro, Jinju, Korea; Departments of Laboratory Medicine, Gyeongsang National University School of Medicine, 79 Gangnam-ro, 660-702 Jinju, Korea; Institute of Heath Sciences, Gyeongsang National University School of Medicine, Jinju, Korea; Green Cross Laboratories, Yongin, Korea

**Keywords:** *PDGFRB*, *TRIP11*, Therapy-related myeloid neoplasm, Acute promyelocytic leukemia, t(5;14)(q33;32)

## Abstract

**Background:**

Therapy-related myeloid neoplasm after treatment for acute promyelocytic leukemia (APL) is a relatively infrequent but severe complication. Most therapy-related myeloid neoplasms after treatment for APL are classified as therapy-related myelodysplastic syndrome or therapy-related acute myeloid leukemia. Translocation of 5q31-33, *PDGFRB* occur rarely in therapy-related myeloid neoplasm and there has been two identified *PDGFRB* partner genes located at 14q32, *TRIP11* and *KIAA1509*.

**Results:**

The *TRIP11-PDGFRB* fusion was identified in a patient with therapy-related myeloid neoplasm with t(5;14)(q33;q32) after treatment of APL using conventional cytogenetics, fluorescence in situ hybridization (FISH) and molecular methods. Cytogenetic analysis of the bone marrow aspirate revealed 46, XY, t(5;14)(q33;q32) in all 20 analyzed cells. No other cytogenetic abnormalities were observed. Break-apart FISH analysis demonstrated that rearrangement of *PDGFRB* at 5q33 was positive in 460 of 500 cells analyzed, while the *PML-RARA* rearrangement remained undetectable by RT-PCR. Sequencing of RT-PCR products revealed fusion between exon 16 of *TRIP11* and exon 11 of *PDGFRB*. However, the *KIAA1509-PDGFRB* fusion was not detected by RT-PCR.

**Conclusion:**

We firstly demonstrated that therapy-related myeloid neoplasm with *TRIP11-PDGFRB* fusion was identified after treatment of APL.

## Background

Therapy-related myeloid neoplasms (t-MNs) are late complications of cytotoxic therapies used to treat malignant and non-malignant conditions. The incidence of t-MNs is increasing worldwide, due to improved survival rates following treatment of primary malignancies [1]. t-MNs account for 10-20% of all malignant myeloid diagnoses [2].

The introduction of all trans retinoic acid (ATRA) has been a major breakthrough in the treatment of acute promyelocytic leukemia (APL), characterized by t(15;17)(q22;q12). The combination of ATRA and anthracycline-based chemotherapy results in high rates of complete remission and survival. However, ATRA combined with chemotherapeutic drugs increases the risk of t-MN. Most t-MNs after treatment for APL are classified as therapy related myelodysplastic syndrome (t-MDS) or therapy related acute myeloid leukemia (t-AML). Rearrangement of platelet-derived growth factor receptor beta (*PDGFRB*) is a distinctive type of myeloid neoplasm which occurs rarely in t-MN. More than 20 different partner genes of *PDGFRB* have been described [3]. The most common abnormality is the t(5;12)(q31 ~ 33;p12), which forms an *ETV6-PDGFRB* fusion gene. The *TRIP11-PDGFRB* fusion gene and the *KIAA1509* (HGNC approved gene symbol; CCDC88C)*-PDGFRB* fusion gene were identified in a patient with myeloproliferative neoplasm (MPN) and t(5;14)(q33;q32) [4,5]. t-MN has an extremely poor clinical outcome. However, t-MNs with *PDGFRB* rearrangement are very sensitive to imatinib methylate, a tyrosine kinase inhibitor with activity against *ABL*, c-*KIT*, and *PDGFR* [6].

Here, we report the detection of a *TRIP11-PDGFRB* fusion in a patient with t-MN with t(5;14)(q33;q32) after treatment of APL.

## Case presentation

**Figure 1:**
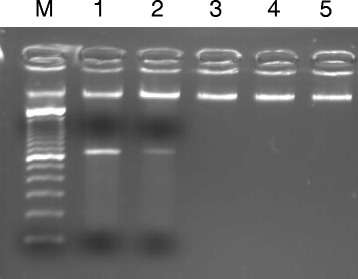
**RT-PCR using primers located in** ***TRIP11*** **exons 14-16 and** ***PDGFRB*** **exons 11-12 resulting in amplification of** ***TRIP11-PDGFRB*** **fusion transcripts.** M: Molecular weight marker, lane 1; 5′ *TRIP11* and 3′ *PDGFRB* specific primers (patient), lane 2; Replication of 5′ *TRIP11* and 3′ *PDGFRB* specific primers (patient), lane 3; 5′ *TRIP11* and 3′ *PDGFRB* specific primers (control), lane 4; 5′ *KIAA1509* and 3′ *PDGFRB* specific primers (patient), lane 5; 5′ *KIAA1509* and 3′ *PDGFRB* specific primers (control).

**Figure 2:**
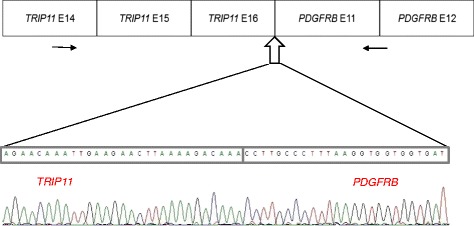
**Sequence chromatogram of the** ***TRIP11-PDGFRB*** **fusion junction showing the fusion between exon 16 of** ***TRIP11*** **and exon 11 of** ***PDGFRB*** **.** Black arrows indicate the location of primers. The white arrow indicates the location of breakage and reunion. E: exon.

A 54-year-old Korean male was referred to our hospital due to hematemesis in July of 2010. Peripheral blood results showed a hemoglobin of 9.1 g/dL, a platelet count of 23 × 10^9^/L, and a leukocyte count of 1.3×10^9^/L with 30% abnormal promyelocytes. The bone marrow aspirates and biopsy showed hypercellular marrow with 95% abnormal promyelocytes, including faggot cells. Karyotypic analysis of the bone marrow revealed 46, XY, t(15;17) (q22;21) in all 20 metaphases that were analyzed. No other cytogenetic abnormalities were observed. The *PML-RARA* fusion gene was detected using RT-PCR and FISH analysis. A diagnosis of acute promyelocytic leukemia with t(15;17) (q22;21);*PML-RARA* was made based on the World Health Organization (WHO) 2008 classification of myeloid lymphoid neoplasms. The patient was treated with ATRA in combination with idarubicin. The presence of the *PML-RARA* rearrangement was monitored by FISH and quantitative RT-PCR. He achieved a complete remission. The patient received consolidation and maintenance chemotherapies. He was monitored at regular time intervals and no *PML-RARA* was detected in the bone marrow after consolidation and maintenance therapies. At a regular follow-up analysis in October 2013, peripheral blood results showed a hemoglobin of 12.3 g/dL, a platelet count of 104 × 10^9^/L, and a leukocyte count of 19.8×10^9^/L, with 58% neutrophils and 5% eosinophils. The bone marrow aspirates showed myeloid hyperplasia with eosinophils and eosinophil precursors (13%) without blast excess (1%). Karyotypic analysis of the bone marrow revealed 46, XY, t(5;14)(q33;q32) in 20 metaphase cells. The *PDGFRB* rearrangement was detected by FISH analysis, while the *PML-RARA* rearrangement remained undetectable by RT-PCR. Since there has been two identified *PDGFRB* partner genes located at 14q32, *TRIP11* and *KIAA1509* (http://atlasgeneticsoncology.org), RT-PCR analysis was performed to specify the partner gene. As a result, *TRIP11-PDGFRB* fusion transcript, not *KIAA1509- PDGFRB* was detected (Figures 1 and 2). A final diagnosis of t-MN with t(5;14)(q33;q32) and a *TRIP11-PDGFRB* rearrangement was made.

## Methods

### Conventional cytogenetic analysis and FISH analysis

Chromosomes were analyzed with GTG-banding and the karyotype was described according to the International System for Human Cytogenetics Nomenclature, 2013. FISH analysis was conducted using a dual color *PDGFRB* breakapart probe (5q33, D5S1907, centromere side, red signal/D5S2014, telomere side, green color). The manufacturer’s recommendations were followed for the hybridization and post-washing procedures (Abbott Molecular/Vysis, Des Plaines, IL, USA). The expected normal signal pattern is two red/green fusion signals. When cells have *PDGFRB* gene rearrangement, the fusion signal would be split into a red and a green signal.

### Molecular methods

To determine whether *TRIP11* or *KIAA1509* were fused to *PDGFRB*, RNA extraction was performed with the Qiamp RNA Blood Mini Kit (Qiagen, Hilden, Germany). Reverse transcription was performed with random hexamers and MMLV reverse transcriptase (Life Technologies, NY, USA). RT-PCR was performed with Takara LA Taq DNA polymerase (Takara Bio Inc, Shiga, Japan). For the detection of fusion transcript between *TRIP11* and *PDGFRB*, 5′ *TRIP11* and 3′ *PDGFRB* specific primers were used as previously described [4], using 1407CF (5′-CGCTGCAGCTTTCTGTCTCTCAGGAACAAG-3′) and 2022PR (5′-GTAACGTGGCTTCTTCTGCCA-3′). RT-PCR was performed with ABI 2720 (Life Technologies, Foster City, CA, USA) and cycles were as follows: initial denaturation at 94°C for 1 min; then 35 cycles of 94°C for 30 sec, 60°C for 30 sec and 72°C for 2 min; and a final extension of 5 min at 72°C. Direct sequencing of amplified products was performed with ABI 3130 (Life Technologies). Sequences were analyzed using Sequencher software (version 4.10 Gene Codes Corporation, Ann Arbor, MI, USA). For the detection of a fusion transcript between *KIAA1509* and *PDGFRB*, 5′ *KIAA1509* and 3′ *PDGFRB* specific primers were used as previously described [5], using *KIAA1509-RTF1* (5′-ccgggacacagataagac-3′) and *PDGFRB-RTR1* (5′-catgatcttcagctccgaca-3′).

## Results and discussion

We have identified a *TRIP11-PDGFRB* fusion in a patient with t(5;14)(q33;q32) after treatment for APL. Cytogenetic analysis of a bone marrow aspirate revealed 46, XY, t(5;14)(q33;q32) in 20 analyzed cells. Break-apart FISH analysis demonstrated that rearrangement of *PDGFRB* at 5q33 was positive in 460 of 500 cells analyzed. Sequencing of RT-PCR products revealed fusion between exon 16 of *TRIP11* (NM_004239.3) and exon 11 of *PDGFRB* (NM_002609.3), which is the same breakage and reunion point as that reported in the previous study [4]. The *KIAA1509-PDGFRB* fusion gene was not detected by RT-PCR (Figures 1 and 2). t-MN after treatment for APL is an infrequent but severe complication. The incidence of t-MN was 1% - 6.5% [7,8]. t-MDS is a more common complication than t-AML when considering t-MNs [7-9]. The most common cytogenetic abnormalities were partial and complete deletions of chromosomes 5 and 7, and 11q23 rearrangements. Translocation of 5q31-33 and *PDGFRB* occur rarely in t-MN. The *PDGFRB* gene at 5q33 is normally expressed in erythroid and myeloid precursors in the bone marrow, and it is a receptor tyrosine kinase which plays an important role in wound healing and other processes in adults [10,11]. *PDGFRB* is disrupted by other translocations, such as t(5;12), or t(5;14). The most common abnormality is t(5;12)(q31-33;p12), which forms an *ETV6-PDGFRB* fusion gene. The *ETV-PDGFRB* fusion gene was identified in patients with chronic myelomonocytic leukemia (CMML) and translocation t(5;12)(q33;p13) by Golub and Gilliland in 1994 [12]. *ETV-PDGFRB* is a chimeric tyrosine kinase protein which is constitutively active due to enforced homodimerization by self-association domains present on the fusion partner protein (*ETV6*). This in turn activates downstream signaling pathways, resulting in cell proliferation and survival [13]. Levin et al. cloned *KIAA1509* as a *PDGFRB* fusion partner in imatinib-responsive myeloproliferative disease associated with t(5;14)(q33;q32)[5]. Expression of the *CEV14-PDGFRB* fusion gene has been reported in acute myeloid leukemia with t(7;11) after clonal evolution [4] in one reported case of t-MN after treatment for AML. Abe et al. hypothesized that the *CEV14-PDGFRB* fusion causes ectopic constitutive tyrosine kinase activation of *PDGFRB*, leading to transformation via the *ras* signal transduction pathway [4]. *CEV14* is homologous to *TRIP11*, which was isolated as one of the proteins that interacts with thyroid hormone receptors in the presence of T3. In our case, response to therapy was monitored by FISH and RT-PCR for the *PML-RARA* rearrangement. At his last regular follow-up, RT-PCR for *PML-PARA* was negative, and eosinophilia was mild. Eosinophilia has been reported in recurrent chromosomal aberrations. 5q31-33 is often involved in myeloid disorders with eosinophilia, suggesting involvement of a *PDGFRB* rearrangement. t(5;14)(q33;q32) was detected with conventional cytogenetics and FISH analysis for *PDGFRB* was positive. In order to identify partner genes of *PDGFRB*, such as *KIAA1509* or *TRIP11*, we performed RT-PCR and direct sequencing for both. We have identified a *TRIP11-PDGFRB* rearrangement. Detection of *PDGFRB* rearrangement is important because these rearrangements respond well to treatment with imatinib mesylate [6].

The majority of abnormalities involving *PDGFRB* are detected by conventional cytogenetics. Performing conventional cytogenetics on a regular basis in all treated APL patients for the early detection of t-MNs implied by chromosomal aberrations is important.

## Conclusion

This is the first report of t-MN with a *TRIP11-PDGFRB* rearrangement after treatment of APL.

## Consent

Written informed consent was obtained from the patient for publication of this Case report and any accompanying images. A copy of the written consent is available for review by the Editor-in-Chief of this journal.
